# Public Health in a Federation: Lessons from the Spanish Influenza in Australia

**DOI:** 10.1093/shm/hkac005

**Published:** 2022-02-09

**Authors:** Carolyn Holbrook

**Keywords:** public health, public health and quarantine, Spanish influenza in Australia, public health and federalism, public health and nationalism

## Abstract

Public health policy has been identified by scholars as a principal means by which the state has expanded its control over human populations. Yet, as the COVID-19 pandemic has shown, public health responses do not necessarily reinforce the authority and prestige of the state, even while governments employ strict measures such as lockdowns and border closures. This article examines arguments about the nation-making effects of public health measures through an examination of the Spanish influenza outbreak in the recently federated Australian nation during 1919. It examines the effort of the central government to co-ordinate quarantine and other public health measures in the face of serious tensions within the Australian federation. In doing so, the article suggests a need to think more subtly about the role of ‘bio-political’ events such as public health crises in consolidating state control and fostering exclusionary forms of nationalism. These lessons apply particularly to federal nation-states.

One of the many notable phenomena since the COVID-19 pandemic began is the wildly varied capacity among nations to protect their citizens from the virus. Various commentaries have observed the lack of obvious commonalities of governance or affluence among those countries that have achieved low rates of infection and mortality.^1^ Some democratic nations, such as South Korea, Taiwan, New Zealand and Australia, have kept mortality rates at comparatively low levels. Others, most notably the USA, the UK and India, have been far less effective at preventing loss of life. China’s authoritarian government has done markedly better than many democratic nations, but another one-party state, Russia, has fared less well.^2^

Federalism, according to an article published in the *Journal of Health Politics, Policy and Law* in late 2020, has hindered the USA’s response to the novel coronavirus. The article claimed that the American federation, which disperses sovereignty between the national and state governments, has caused conflict and confusion concerning such crucial matters as physical distancing, testing and the use of masks and ventilators.^3^ Overall, the authors concluded: *‘*This fragmented and disjointed response has undoubtedly cost time and lives’.^4^ A similar argument has been made in the Australian context, where public health powers are also divided between central and state governments. The long-time health adviser Bill Bowtell, who led Australia’s lauded policy response to the AIDS/HIV pandemic in the 1980s, blamed ‘deep fault lines in the outmoded structures of the Australian federation’ for substantial shortcomings in the nation’s response to COVID-19. Of greatest concern to Bowtell were the failures of co-ordination between state and federal authorities, which led casual workers to seed a second wave of the virus that raced through Melbourne’s aged care homes during the southern hemisphere winter of 2020.^5^ Bowtell claimed that Australia’s federal system (derived from that of the USA) ‘has become a dead weight around the neck of Australia’s democracy and people’.^6^

Citizens of Western Australia, Australia’s largest and most isolated state, would presumably disagree with Bowtell’s assessment. The state’s Labor premier, Mark McGowan, took a hard-line approach to pandemic control, closing the interstate border for most of 2020, despite the protestations of the prime minister and other states. While Victorians endured a 3-month lockdown in mid-2020, and New South Wales and Queensland dealt with sporadic outbreaks of COVID-19, the lives of Western Australians continued in relative normality. Premier McGowan was rewarded with an historic election win in March 2021, which reduced the principal opposition Liberal Party to just two seats in the Legislative Assembly. His willingness to close the border and pit Western Australia against the ‘eastern states’ Goliaths tapped into long-standing and deeply held secessionist sentiment within Western Australia.^7^

McGowan’s actions in 2020–21 recall those of another West Australian premier during the Spanish influenza pandemic 100 years earlier. During that public health emergency, the unity of the newly created Commonwealth of Australia rapidly fragmented as conflicting subnational interests and mismanagement by the central government subverted attempts at a co-ordinated national approach. This article will explore the issue of pandemic disease governance in a federal body politic, using the case study of Australia during the Spanish influenza pandemic of 1918–19. Through an examination of state and central government responses to the pandemic, I will argue that historiographical understanding of the relationship between public health practices and state authority needs to be more sensitive to the nuances of the federal body politic. This argument also has implications for how governments seek to manage the COVID-19 pandemic and future infectious disease outbreaks.

## Public Health in Early Twentieth-century Australia

Public health policy has been identified by scholars as a means by which the state has expanded its control over human populations.^8^ Over the centuries, the state has responded to outbreaks of bubonic plague, smallpox, cholera and other diseases with a raft of public health measures that include quarantine, cordons sanitaire, lazarettos, sanitoria, urban renewal programmes and vaccination. These measures have, according to the historian Frank M Snowden, ‘justified control over the economy and movement of people; they have authorised surveillance and forcible detention; and they sanctioned the invasion of homes and the extinction of civil liberties’.^9^

Michel Foucault’s pioneering work on anatomo politics, bio-power and bio-politics described the role of the modern state and the medical profession in harnessing new technologies to discipline and punish individuals and to control populations.^10^ Supervision of processes related to the protection of human life—birth and death, procreation, health and life expectancy ‘was effected through an entire series of interventions and *regulatory controls: a biopolitics of the population*’.^11^ Foucault linked the emergence of these networks of bodily control at the individual and collective levels in the seventeenth and eighteenth centuries to the rise of modern capitalism. The new discourses and their regulatory apparatus made possible: ‘the controlled insertion of bodies into the machinery of production and the adjustment of the phenomena of population to economic processes’.^12^ Public health can thus be conceived as a ‘bio-political’ phenomenon, which can be used to control populations and reinforce the demarcation between those who belong within the nation-state and those who must be excluded. As Alison Bashford has summarised, ‘health’ has become central to modern national identity, through ideals such a healthy population, a nation of healthy citizens, national fitness, national hygiene and the prevention of harms through border control.^13^

Delineations and identities created in the name of public health have often been drawn in racialised terms. Much of the Australian research has described how social Darwinian and eugenicist concepts of race were used to control Indigenous peoples and non-white migrants.^14^ In his landmark study, *The Cultivation of Whiteness* (2002), the historian Warwick Anderson demonstrated the ways that medicine and public health have acted as instruments of British settler colonialism.^15^ Anderson shows how the Australian tropics became a site through which anxieties about racial fitness were mediated, and how the settler project was enveloped in and legitimated by the exalted rhetoric of science. Using the insights of critical race theory and whiteness, Christopher Mayes has argued that the establishment of the Australian state rested on a pact between democratic liberalism and racism. Mayes argues that whiteness was a norm around which pervasive and enduring epistemological and institution foundations were built.^16^ Historians have also observed the parallels that were drawn in the early twentieth century between infectious diseases and other external ‘threats’, such as communism and Asian invasion.^17^ The Commonwealth of Australia, formed in 1901, responded to these perceived threats in ways that included not only quarantine and other public health measures but also citizens’ forces, cadet training and physical fitness programmes.^18^

Historians of public health have described the categories of race and mental capacity that Australian migration policy imposed in the early twentieth century.^19^ Of particular relevance to the Spanish influenza is Alison Bashford’s work on the nexus between infectious disease control and racialism, part of her larger contribution to understandings of quarantine, racial hygiene and, more recently, population. Bashford conceived the Australian quarantine system, not merely as a practice for excluding disease, but as an act of spatial imagination, which is deeply embedded in the modern process of nation-making:

Boundaries are required for the creation of nations in a modern Western sense, and quarantine is in essence the putting of these boundaries to a particular use by the administrative nation-state. Not infrequently, quarantine and national administration produce and monitor the same space: that is, the border of a nation has often been where a quarantine line was drawn. This same border might well have a military, political and economic significance; the place of potential invasion and defence as well as commercial traffic and exchange.^20^

Bashford argues that Australian quarantine practices have been ‘important to the creation of a sense of national boundary’.^21^ Echoing the nation’s White Australia immigration policy, which effectively barred the entry of non-white migrants, quarantine practices defined the racial borders of the nation-state. Bashford claimed that the power over quarantine that was given to the Commonwealth (central) government at the time of Federation in 1901 became the crucible of a larger public health project that defined and administered the Australian nation in racialised terms.^22^

Though there is a rich Australian historiography of Spanish influenza, it has not addressed directly the thesis advanced by Bashford about the nexus between the quarantine power and racialised nation-making. Much of the research about the Spanish influenza in Australia takes a regional perspective and employs social and oral history methodologies.^23^ Some scholars have addressed the Spanish influenza as part of the history of quarantine stations, while others have considered its place in the national memory. Where historians do take a national perspective, however, they appear to contradict Bashford’s representation of quarantine as a homogenising process. They conceive the pandemic as an event that demonstrated the limits of central power and indicated the brittleness of the compact that was created in 1901 when the six British colonies of New South Wales, Victoria, Queensland, South Australia, Western Australia and Tasmania federated to become the Commonwealth of Australia.^24^ Humphrey McQueen observed that Australians had embraced the new ‘consciousness of nationhood’ spurred by their participation at Gallipoli and in other campaigns during the First World War; a sentiment that was expressed in the emerging Anzac legend.^25^ Indeed, the war was widely heralded as the ‘birth of the nation’, a moment that Federation in 1901 had conspicuously failed to induce.^26^ McQueen argued, however, that Australians lacked a civic-minded patriotism that could summon the unity and common purpose required to protect citizens from the influenza virus:

In 1915, an external menace had driven Australians together; by 1919, an internal danger revealed yet again how easy it was for Australians to stand apart. If national unity involved loyalty to the Commonwealth as an administrative machine, the Pandemic showed how little of it there was.^27^

While historians have noted the administrative failures of the Commonwealth and the disunifying effects of the Spanish influenza, there has been no detailed examination of federal relations during the pandemic. Nor has there been consideration of what this fracturing of national sentiment implies for our understanding of public health practices. In what follows, I will outline the attempts of the Commonwealth government to stake a commanding role in Australian public health, and detail how the Spanish influenza pandemic undermined these centralising efforts.

## Public Health in the Early Australian Commonwealth

Europeans in Australia enthusiastically adopted the maritime quarantine practices that had been used for centuries to control the spread of infectious diseases such as bubonic plague and smallpox in other parts of the world.^28^ New South Wales enacted quarantine legislation in 1832 in response to the threat of European cholera and operated a permanent quarantine station at North Head from 1837.^29^ Victoria established a quarantine station at Point Nepean in 1852 and other colonies followed suit. The Australian colonies continued to develop and refine their quarantine practices, even while they were falling from favour in Europe and North America.^30^ Amid the growing belief that quarantine was a blunt instrument with dire economic consequences, in 1825 a British parliamentarian condemned it as ‘anti-commercial, anti-social and anti-Christian’.^31^ Quarantine was further discredited when it failed to prevent the cholera epidemic of 1866–67.^32^ By 1873, Britain had abandoned quarantine altogether, in favour of sanitary measures and medical surveillance.^33^ Ships suspected of carrying disease were inspected and those found to be infected were removed to isolation hospitals.^34^ Other passengers were released but regularly inspected by medical officers until the risk of infection passed.^35^ The historian Krista Maglen claims that the Australian colonies departed from what became known as the ‘British model’ of disease prevention for three reasons: their public health infrastructure was less developed, which meant the sanitary approach would be less effective; vaccination was less available as a treatment option, and Australia remained free from some diseases that were endemic in other parts of the world.^36^

Following the mid-century discovery of gold in Victoria, shipping traffic greatly increased to the Australian colonies, and with it the threat of infectious disease.^37^ The desire to streamline quarantine practices became an incentive for improved administrative co-operation between the Australian colonies.^38^ The Medical Adviser to the government of New South Wales, Charles Mackellar, advocated a federal system of quarantine in1883, claiming that consistent practices, jointly administered by the colonies, were vital ‘for our common weal’.^39^ Though a mooted ‘Federal Quarantine Act of Australasia’ never materialised, quarantine was the single public health power granted to the Commonwealth in 1901.^40^ The colonies did not contest the transfer of quarantine power to the Commonwealth, though the future Chief Justice of the High Court, R.E. O’Connor, wondered whether the power should be limited to maritime borders, as federal control of internal quarantine might conflict with existing state jurisdiction over matters of public health.^41^

Several outbreaks of plague in the years after Federation prompted the Commonwealth to enforce its constitutional power over quarantine.^42^ Following the passage of *An Act Relating to Quarantine* in 1908 and the establishment of a Federal Quarantine Service in 1909, quarantine powers were transferred from the states to the Commonwealth. These included power over internal quarantine, despite the objections of Western Australia and New South Wales.^43^ Dr John Howard Lidgett Cumpston, a leading figure in the burgeoning field of public health, became acting director of the Quarantine Service in May 1913 and permanent director a couple of months later. Melbourne-trained Cumpston had observed directly the American physician Victor Heiser’s success in eliminating cholera and smallpox in the Philippines and was resolved to protect Australia from ‘the contagious diseases of the East’.^44^ Cumpston was internationally connected; he had earned a Diploma of Public Health in London and had links with leading experts in bacteriology, hygiene and public health in Paris, Brussels and Berlin.^45^

Cumpston was prominent in the Australian expression of a wider, international push for greater state intervention to combat ill-health. He envisioned that:

the proper objective of governmental administration was much greater than the prevention and control of epidemics; that it was, in fact, nothing less than positive health, freedom from all illness and disability for every individual human unit in the community.^46^

The new Director of Quarantine had a difficult start in his new role. Following an outbreak of smallpox in Sydney in July 1913, he used his authority to restrict the movement of people to and from parts of the city, and to require people leaving Sydney to be vaccinated. Cumpston was criticised by the New South Wales government, health officials and the press, which perceived his actions as unwarranted Commonwealth interference. Cumpston reflected later that the episode taught him ‘the impossibility of reconciling dispassionate decisions based upon technical knowledge with impetuous political actions’.^47^

The historian Michael Roe placed Cumpston in the vanguard of a group of Australian progressives in the early twentieth century for whom ‘the notion of improved public health blended with aspirations towards racial perfection and physical excellence’.^48^ Along with contemporaries such as Dr J.S.C. Elkington and Dr Richard Arthur, Cumpston grafted rapidly emerging scientific knowledge about the causes of ill-health to less rigorous ideas drawn from social Darwinism that encouraged competition among the ‘races’.^49^ As the Queensland Home Secretary announced upon the passage of the *Queensland Hospital Act* in 1923: ‘Today the health of the individual is a matter of community interest’.^50^ Emphasis was placed on the maintenance of ‘racial hygiene’ through the measurement and physical education of school children. Workers were conceived as ‘industrial units’, whose good health contributed to the ‘wealth of the State’.^51^

The British Medical Association of Australia (BMA) and the influential *Medical Journal of Australia* were sympathetic to expansion of the state’s role in public health.^52^ The BMA was dismayed by the lack of ‘a national point of view’ on health matters.^53^ It favoured the ‘nationalisation’ of medical services—a deliberately nebulous term—and the creation of a position of Federal Minister for Health.^54^ Such influential support was vital if Cumpston were to successfully flesh out a public health role for the Commonwealth from the bones of its constitutional power over quarantine. The most vexed issue remained how this newly staked out Commonwealth role would complement the responsibility for health policy that was left with the states under the terms of the federal settlement in 1901.^55^

The First World War had given a great boost to the cause of centrally co-ordinated public health measures.^56^ For progressive idealists one of the greatest lessons of the First World War was the value of expertise and planning. In his hope-filled template for post-war Australia, *In Your Hands Australia*, Charles Bean wrote:

I do not think that anyone who ever saw that vast organisation behind the lines in France will ever again go back to the theory that the nation can muddle through somehow. The lesson of the war was that by organisation you can do anything … if we want to make Australia the greatest and best country, it can be done by planning. If the plan and the scheme is perfect enough; and if the thousands of brains that we can bring to bear on the subject are bright and active and energetic enough, we can make our country as near perfect as it is possible for a country to be. ^57^

This war-inspired confidence extended to public health reform.^58^ Cumpston expected men to return from the war with a new maturity, confidence and willingness to work co-operatively to achieve national benefit, because a new-found ‘national spirit’ had been awakened by the war.^59^ With the resolute application of planning and expertise, problems such as hospital provision, infant mortality, tuberculosis and venereal disease could be alleviated. There were already examples to draw upon. In 1916, faced with a shortage of diphtheria and tetanus sera, the Hughes government established the Commonwealth Serum Laboratories (CSL) in Melbourne to manufacture and distribute sera not otherwise available. The Commonwealth had committed to subsidising the efforts of state governments in controlling venereal disease and to assisting with an experimental anti-tuberculosis programme in Bendigo.^60^ Victor Heiser, now working for the Rockefeller Association’s International Health Board, negotiated a joint project between the Commonwealth and the Queensland government to assist in the eradication of hookworm, with the hope that such an arrangement would extend to other states.

The nation had passed the public health tests raised by an extra-territorial war with flying colours, but the optimism of reformers was soon deflated by bureaucratic hurdles in the domestic arena. Victor Heiser had hoped to unburden more Rockefeller largesse during his 1916 visit but found that the ‘Federal authorities were not sufficiently powerful to compose State differences and embark on a national health programme’.^61^ In his 1936 memoir, Heiser recalled that: ‘Warfare of one kind or another was always being waged against a neighbouring city, State, or even the Federal Government. Because each State considered itself autonomous, inter-State jealousy, political and economic, was intense’.^62^ Just as the war was ending, the Spanish influenza would visit a major health crisis on Australian shores and test the capacity of the restive young Federation in new ways.

## Maritime Quarantine

The source of the 1918–20 Spanish influenza pandemic, as it was most commonly called in Australia, remains a matter of contention. The disease appeared in American military training camps in Kansas in March 1918 and in the French military base of Brest in early April that year.^63^ Wherever its origin, by June 1918 it had spread over continental Europe and North America, and to Britain, China and India. After the initial surge of infections subsided, a more aggressive second wave devastated Europe, America, parts of Africa and Asia in July–August 1918. This wave was highly infectious with a far higher mortality rate than seasonal flu. And, unlike seasonal flu, which was most dangerous to infants, the elderly and the vulnerable, the Spanish flu disproportionately affected healthy young men and women.

The greatest danger to Australia was posed by ships carrying troops returning from Europe. Maritime quarantine—indisputably a matter of Commonwealth prerogative—would be the first line of Australian defence. During the second week of October 1918, there was a severe outbreak in South Africa and, soon after, in New Zealand.^64^ The Commonwealth responded by requiring that ships that had been in contact with South African or New Zealand ports undergo 7 days in quarantine. As Director of Quarantine, Cumpston sought to reassure the public ‘that we are strongly and efficiently equipped right throughout the Commonwealth to deal with the scourge’.^65^ Sydney and Perth bore the brunt of quarantine operations; by mid-November, New South Wales had quarantined between 2,500 and 3,000 people and Western Australia between 2,000 and 3,000. Victoria had managed between 800 and 900 passengers, South Australia between 500 and 600, and Tasmania, just 50.^66^ By the time that quarantine restrictions were eased in April 1919, 228 vessels had been detained, 18,697 crew and 55,147 passengers.^67^

As Richard O’Connor had foreseen during the Federation debates, the Commonwealth’s jurisdiction over internal quarantine clashed with state authority over land use and public health. An early point of controversy emerged when Commonwealth officials declined the admission of Catholic priests to the North Head quarantine station to administer last rites to dying patients. When the New South Wales government backed the Catholic Church, the Commonwealth relented. There was also much consternation in New South Wales about the proximity of the North Head quarantine station to the populous suburb of Manly. The doctor and social reformer, Richard Arthur, instigated a motion in the Legislative Assembly in November 1918, arguing that the station should be relocated from the vicinity of a major population centre to Jervis Bay.^68^ Though located within New South Wales, Jervis Bay had become a Commonwealth territory in 1915. Arthur and others in the New South Wales parliament engaged in acrimonious outpourings against the Commonwealth. One member claimed that federal politicians: ‘think themselves superior to the States, and that matters emanating from the State Parliaments should rarely, if ever, be taken notice of’.^69^ Another complained that:

The interests of New South Wales should not be treated as a mere bagatelle. There is a tendency on the part of the Federal authorities to treat our interests as of no concern because we are only a little place 500 miles away from Melbourne, the centre of Government. This question involves the health and lives of the whole of the people in New South Wales, and yet we have to submit to the domination and dictation of a few people in Melbourne.^70^

Cumpston’s allies at the *Medical Journal of Australia* sprung to his defence. The journal’s editorial condemned Arthur’s motion that the North Head quarantine station be closed, and called for a national approach to public health: ‘It is a great pity, after four years of war, fighting side by side, not as members of detached States, but as citizens of a great Commonwealth, that a prominent politician should exhibit signs of such desperation’, it read.^71^ The journal accused state politicians of seeking to resist the legitimate authority being exercised by the Commonwealth:

It is not unnatural that those who realize the inevitable superseding of the Federal control for State control in matters of communal interests, and who have failed to grasp the real significance of an Australian nation, should manifest jealousy at the complete success of the quarantine measures adopted by the Federal authorities …^72^

West Australians were equally dissatisfied with Commonwealth quarantine arrangements. Following outbreaks of Spanish flu on returning troop ships, there had been public outcry about the poor conditions endured by both the soldiers and the nurses who tended them at Woodman’s Point, south of Fremantle.^73^ Feelings intensified when 600 soldiers were forced to remain on the highly infected *Boonah* because Woodman’s Point was overcrowded. At public meetings and in newspaper correspondence, West Australians condemned the treatment of the soldiers, who had been ‘willing to sacrifice so much’ in the war.^74^ Protesters delineated between the state and federal spheres, and directed their ire at a Commonwealth government that was ill prepared for the influx of ships. The state Minister for Public Health, Hal Colebatch, made sure to emphasise that quarantine was a matter for the federal government: ‘Every day makes more clear the futility of control from Melbourne in a matter of this kind’, he told the press. ‘The exact local knowledge and prompt decision founded thereon are the two essentials. With control from Melbourne we have neither of these.’^75^

Despite clashes between the Commonwealth and the states, there was also admiration for Cumpston’s maritime quarantine programme. It appeared that the disaster that had unfolded in South Africa and New Zealand had been averted in Australia. By December 1918 Cumpston felt confident to pronounce that ‘the period of greatest danger was certainly over, though constant watchfulness would continue to be exercised’.^76^ A correspondent to the *Medical Journal of Australia* offered his ‘warm congratulations’ to the director and officials of the Quarantine Department: ‘While we were threatened with this dire epidemic, they saved the whole of Australia’, he wrote.^77^ The *Medical Journal of Australia* judged that: ‘The Federal authorities [had] attacked this problem of excluding a highly infectious disease from the Commonwealth with energy, skill and intelligence’. Not missing a chance to push its case, the editorial noted that ‘the thoroughness of their work must be regarded as one of the strongest arguments in favour of the abolition of State control, and the substitution of Federal control in all matters concerning the public health’.^78^

## The November Agreement and the Premiers’ Conference

Though the maritime defence remained intact, the Commonwealth decided in November 1918 to devise a plan of action with the states in the event that the disease escaped quarantine. State ministers of health, directors of state health departments and branch presidents of the British Medical Association were invited to confer with the Minister for Trade and Customs, Walter Massy-Greene and the Director of Quarantine in Melbourne. The location prickled New South Wales sensitivities; the acting premier requested that proceedings be moved northwards to the ‘storm centre’ of the disease.^79^ The meeting proceeded as planned in Melbourne on 26 November, where the disease was officially designated ‘pneumonic influenza’, in light of its capacity to cause severe secondary infection of the lungs.^80^ The conference produced a 13-point plan of action. Each state agreed to establish an advisory committee consisting of the chief health officer and members of the British Medical Association. Other preparatory measures included the establishment of vaccine depots and special hospitals and arrangement for ambulance transport, respirators and medical and nursing assistance in the event of an outbreak. The states agreed to provide advice to local authorities through the press and by circular.^81^

Massy-Greene and Cumpston were sceptical of the value of border quarantine within mainland Australia—except in the case of Western Australia, which was sufficiently isolated for the strategy to have some merit.^82^ The premiers insisted, however, that movement be checked as far as possible in the event of an outbreak.^83^ Restrictions on travel would buy neighbouring states time to complete their preparations and ‘afford a delay which may result in some diminution of the virulence of the epidemic’.^84^ Delegates agreed a clear procedure in the event of pneumonic influenza breaching quarantine. When a case was recognised, the chief health officer of the state would notify the Commonwealth Director of Quarantine. The Commonwealth would then proclaim the state to be infected with pneumonic influenza and close its borders with clean states. Public places such as theatres, musical halls, picture shows, race meetings, churches and schools would be closed. Upon the proclamation of a state as infected, the federal government would take complete control of all interstate traffic by land and sea in order to enforce the quarantine. Under this arrangement, the states were required to ‘render to the Commonwealth every possible aid and cooperate in the effective carrying out of the Regulations’.^85^ Cumpston later characterised the Commonwealth’s assumption of border control with state co-operation as ‘a general affirmation of acceptance of dominant Commonwealth authority’.^86^ If influenza was discovered in a neighbouring state, the border would be reopened. The power to repeal a proclamation that a state was infected would also rest with the Commonwealth. Special dispensation to travel to an infected state could be granted via a permit issued by the Commonwealth government.

As the new year dawned, it looked increasingly likely that the November agreement would be redundant. The pandemic that was claiming millions of lives around the world had not penetrated Australia’s maritime border. The Premiers’ Conference that was scheduled for 22–27 January was due to proceed. Prime Minister Billy Hughes had left Australia in April 1918 to call on American President Woodrow Wilson before sailing to Britain, where he attended meetings of the Imperial War Cabinet. The prime minister remained in Europe to attend the Versailles Peace Conference in early 1919 and didn’t return to Australia until August that year. Hughes had left the Treasurer William Watt in charge. Watt had previously been premier of Victoria and was considered highly capable and a likely successor to Hughes. He had opposed Labor’s referendum proposals to increase Commonwealth powers in a range of social and economic areas in 1911 and 1913 and was regarded as an advocate of states’ rights.^87^

The principal purpose of the conference was to consider the financial discontents that had bedevilled the Federation since its foundation. The most contentious issue remained, as ever, the amount of customs and excise duty returned by the Commonwealth to the states. The states had ceded their right to collect these taxes to the Commonwealth government at Federation in 1901. Faced with massive war debt, the Commonwealth proposed a graduated reduction over 6 years from the current per capita payment of £25 to £10. Watt also wanted to streamline taxation collection and overseas loan raising within the Federation. Soldier repatriation and employment opportunities for returned men were also on the agenda, including a scheme to settle soldiers on the land.

On the back of his quarantine success, Cumpston seized the Premier’s Conference as the moment to press for the establishment of a federal health department. Cumpston’s innocuous-sounding agenda item concerned the co-ordination of Commonwealth and state powers with respect to quarantinable and other diseases.^88^ The Director of Quarantine made a strong case for the Commonwealth to take a more assertive role in health care. Management of the health of soldiers returning with conditions such as venereal disease and tuberculosis could not fairly be left to the states, he argued. The Commonwealth was responsible for the administration of the Northern Territory, Federal Capital Territory and Papua, with New Guinea likely to be added to the list as a result of peace deliberations at Versailles, Cumpston pointed out.^89^ Advances in medical science permitted many of the diseases occurring in these areas of Commonwealth jurisdiction to be successfully treated. The Commonwealth could also co-ordinate responses to diseases such as diphtheria, typhoid, tuberculosis, venereal disease and heart disease among the states: ‘Disease knows no State boundaries, and the efforts of one State may be nullified by the neglect of another’, he wrote.^90^ It was the role of the Commonwealth simultaneously to safeguard citizens from danger and maximise their capacity to contribute to the common good:

Human health controls the defence capabilities of the Commonwealth, determines the working efficiency and therefore the national wealth of the Commonwealth and as, in the final test, all national emergencies are matters for the Commonwealth as a whole human population which determines the capability of the Commonwealth to meet these emergencies should surely be a matter for the earnest attention of the Commonwealth Government.^91^

Cumpston advised his minister to proceed with caution in his dealings with premiers jealous of their constitutional prerogative in matters of public health. Mindful of his bruising experience during the Sydney smallpox outbreak in 1913, he reminded Massy-Greene that: ‘The adjustment of relationships between Commonwealth and States in these matters will require intelligent and careful handling if satisfactory results are to be obtained, and if friction is at the same time to be avoided.’^92^

As the Premiers’ Conference began in the temporary Commonwealth Parliament House on Monday 22 January, rumours spread of an outbreak of pneumonic influenza in Melbourne. These reports had been circulating in medical and political circles for a couple of weeks, only to be stonewalled by the chief health officer, Edward Robertson.^93^ By the third week of January, the superintendent of Royal Melbourne Hospital, Ralph McMeekin, had no doubt that the disease affecting an increasing number of his patients was pneumonic influenza. Growing frustrated by the attitude of health officials, on the evening of 22 January McMeekin announced to the press that there were 27 cases of the disease in the Royal Melbourne Hospital and ‘a good many cases of similar character’ at the Alfred Hospital.^94^ It was not possible to say if this strain was identical to those occurring in New Zealand and in quarantine in Sydney, McMeekin admitted, but ‘Bacteriological examinations’ were being carried out and the Health authorities had arranged for Dr Penfold from the CSL to assist, if required. McMeekin expected to have the results in a day or two.^95^

Cumpston convened an informal conference the following day with McMeekin, Robertson, Penfold, Elkington, who was temporarily in charge of quarantine in Sydney, and another Sydney quarantine doctor, Paul Mitchell.^96^ Cumpston made various claims later in his life about what happened at the meeting on 23 January. In one account he writes that ‘it was recognized and admitted that definite cases of this new form of influenza’ existed at the Melbourne hospital.^97^ In another, he claims that state health authorities acknowledged the presence of the disease after he urged the reluctant Robertson to make an official admission.^98^ For reasons Cumpston does not clarify, this admission did not extend to an official declaration, which would have triggered the arrangements of the November agreement and allowed the Commonwealth to close and control Victorian borders.^99^ But Cumpston’s correspondence and public statements from the time do not match his later versions of events. Far from urging Robertson to confirm an outbreak, Cumpston argued that there was ‘no justification for any alarm’ and that ‘no definite statement’ could be made on the subject until diagnoses were made of the cases under observation.^100^ Cumpston went even further in a statement published in the *Age* on 24 January 1919, claiming the Melbourne cases were *not* Spanish influenza, but a milder version that had been circulating for some time:

The cases reported from various sources have been considered in the light of all the available evidence. It has been decided that, while the cases present an appearance generally similar to some cases that have been dealt with in quarantine, they do not present the same severity of toxaemia, and are not inconsistent with the occurrence of pneumonia, following after influenza of the kind which has been prevalent in Australia for nearly a year.^101^

Robertson, the state’s Chief Health Officer, had a similar message. He told the press on 23 January that there was ‘no proof that the disease was the overseas type’ and that it was ‘premature to enter into any discussion … as nothing definite would be known until late this afternoon’.^102^ Robertson deferred to the results of tests on ‘bacteriological cultures’ drawn from infected patients. To an inquiry from the New South Wales chief medical officer Dr Paton about reports of an outbreak in Melbourne, Robertson replied on 24 January that ‘Bacteriological findings [were] not identical with Sydney cases’ and concluded there was ‘no apparent connection’.^103^

It is tempting to conclude that Cumpston was using the authority of his office and his formidable personality to obtain Robertson’s acquiescence in his tactics of obfuscation and delay—prompted by Cumpston’s own unwillingness to believe that his lauded maritime boundary had been breached. Further, an official notification would trigger the internal border controls, under Commonwealth direction, about which Cumpston and Massy-Greene had expressed disdain. While Cumpston reiterated that official notification remained a Victorian matter, as per the November agreement, he was simultaneously martialling the resources of the Commonwealth to prevent it. Dr Penfold was conducting tests at the CSL, which Cumpston was ‘hopeful’ would indicate the disease circulating in Melbourne was not the strain from overseas.^104^ It was a ‘fortunate coincidence’, Cumpston added, that the Commonwealth officer who had overseen the Sydney quarantine station ‘throughout the whole of the active phase of the true pneumonic influenza there’, was in Melbourne to lend his expertise.^105^

McMeekin was increasingly alarmed by the circumstances at the Royal Melbourne Hospital, which had admitted 14 new cases on Thursday 23 January and was now treating 40 patients.^106^ Others were sent to the Fairfield Infectious Diseases Hospital, when the Royal Melbourne ran out of beds.^107^ On 23 January, McMeekin ‘was emphatic in declaring that the disease was epidemic influenza pneumonia’.^108^ He warned that ‘Every precaution must be taken by the country to prevent the spread of the infection’, including avoiding crowded halls and theatres and forbidding people visiting houses with infected people.^109^ McMeekin equivocated on the question of the disease’s relationship to Spanish influenza; ‘No definite relationship’ could be established, he admitted, but added that the origin of the disease was ‘immaterial from the public standpoint’.^110^

On Friday 24 January 1919, Cumpston and Robertson were summoned to Parliament House by Acting Prime Minister Watt to brief the premiers on the influenza cases in Melbourne.^111^ Cumpston later claimed that he told the premiers that there was ‘no doubt’ the Melbourne Hospital cases were the ‘new disease’, but that he could not act until Victorian authorities made an official notification.^112^ Contemporaneous sources show that his message was unchanged from the day before; it was too early to ascertain whether this was the overseas strain of the disease but that seemed unlikely, he told the premiers.^113^ On the back of this reassurance, the West Australian premier wrote to his deputy in Perth that reports of the outbreak were ‘disquieting’ but he trusted the diagnosis would not prove complaint to be the virulent type.^114^ The New South Wales premier, William Holman, was less mollified, perhaps because he had received a telegram about the Melbourne outbreak from concerned colleagues in Sydney who had convened an urgent meeting of Cabinet. The message reminded Holman of the November agreement and advised that he urge Watt to act on its terms.^115^ When delegates gathered on Saturday morning, Holman’s seat was empty. Fearing that he would be detained in Melbourne if the borders were closed, the New South Wales premier had booked a special train back to Sydney at 10 o’clock that morning.

Cumpston’s bid for an increased Commonwealth role in public health would have to proceed in the absence of the party representing the most populous state. Prime Minister Watt read Cumpston’s memorandum, which outlined two options—the Commonwealth would seek to extend its constitutional authority in order to take a greater role in public health, or it would use existing powers and seek to co-operate with the states and provide expert and financial assistance.^116^ The acting prime minister informed the remaining premiers that he was seeking an indication of their attitudes *before* circulating details to the federal Cabinet. The premiers were not satisfied by this approach. They told Watt that they wished to know whether there was unanimity within the Commonwealth government *before* they considered such a proposal.^117^ In the face of this procedural stalemate, Watt undertook to distribute copies of Cumpston’s memorandum to the states and to convene a special health conference at an unspecified time in the future.^118^

The failure to advance Cumpston’s ambitious scheme for public health characterised a disappointing conference.^119^ Not only had the public health proposal stumbled at its first hurdle, but leaders had been unable to reach agreement on per capita payments, uniform tax collection and loan-raising measures. Cooperation was proving difficult in a system of dual sovereignty in which the federal government was increasingly flexing its financial and administrative muscle. The influenza wave was cresting behind the nation’s political leaders as the Premiers’ Conference concluded on Monday 27 January. The pending epidemic would test the federation in ways that suggested the Commonwealth had not yet learned to yield its expanding authority wisely.

## The Influenza Escapes Quarantine

On Sunday 26 January 1919, Chief Health Officer Robertson, consulted with his advisory committee and reaffirmed his judgement of previous days that the disease afflicting Melburnians was not the same one that was rampant in New Zealand.^120^ Meanwhile, in Sydney a returned soldier who had travelled by train from Melbourne on Tuesday 21 January was displaying ‘suspicious symptoms of pneumonic influenza’ at Number 4 Military Hospital in Randwick.^121^ New South Wales authorities acted quickly. On 27 January, the state declared itself infected, with approximately 20 cases at, and connected to, the Randwick Military Hospital.^122^ The Victorian advisory committee convened again on 28 January and decided that pneumonic influenza did indeed exist in Melbourne.^123^ Victoria declared itself infected on 28 January, the day after New South Wales. The Victorian government ordered the closure of all picture shows, theatres and public halls within 15 miles of the General Post Office, throwing 2,200 men out of employment at a loss of 8,800 pounds weekly in wages.^124^

The consequences of Victoria’s delayed declaration were quickly apparent. By the time that New South Wales declared itself to be infected, there were 420 cases nationally.^125^ Four hundred of those were in Victoria, of which 35 were fatal. All of the 20 or so cases in New South Wales could be traced to people who had recently arrived from Victoria, or their close contacts.^126^ Cumpston was quick to recast his role in the Victorian debacle. He briefed Watt in early February that he had emphasised to Dr Robertson the urgency of obtaining an ‘early and definite decision’ about the nature of the cases, but neglected to mention his own pattern of obfuscation and diversion.^127^

The terms of the November agreement determined that the border between Victoria and New South Wales should remain open because both states were infected. If Victoria had dealt the first blow to the November agreement by delaying its notification, New South Wales landed the second. Correctly perceiving that the southern state was more severely infected, New South Wales issued a proclamation under its own *Quarantine Act* on 29 January, which prevented Victorians from entering New South Wales.^128^ Both Watt and Cumpston expressed ‘the greatest surprise’ at New South Wales’ decision to close the border, while the Victorian premier Harry Lawson called Holman’s action ‘unneighbourly’.^129^ New South Wales began medically inspecting incoming vessels and imposed 4 days of quarantine in addition to the Commonwealth period.^130^ The acting New South Wales premier, George Fuller, exchanged terse messages with Watt about the state’s determination to quarantine ships arriving from Melbourne.^131^ In response to a journalist’s query about whether the Commonwealth was in negotiations with the New South Wales premier, a federal minister replied: ‘I don’t know about negotiations. Perhaps there are recriminations.’^132^

Lawson insisted to Holman that his state had adhered to the ‘spirit and letter’ of the November agreement and suggested that New South Wales, not Victoria, was the source of the influenza outbreak.^133^ Such a claim defied the evidence: by 5 February, Victoria reported 1,452 cases and 79 deaths, while New South Wales had only 56 cases and 1 death. And yet, Holman argued, Victoria claimed the right ‘to send any number of her widely infected population into this State, in which, so far, we have been successful in keeping the disease within small bounds’.^134^ In the absence of Commonwealth action, two quarantine camps were eventually established at the Victorian border, at which land travellers were required to spend 4 days.^135^

Goodwill within the Federation continued to fray as premiers from ‘clean’ states moved to preserve their status. On the afternoon of Monday 27 January, 350 passengers boarded the overnight mail train from Sydney to Brisbane.^136^ At around 10 o’clock that evening, a special issue of the *Commonwealth Gazette* declared New South Wales to be a quarantined area.^137^ Queensland responded by closing its border completely with New South Wales, ignoring an exemption to residents within 10 miles of the border under the November agreement.^138^ The Brisbane-bound passengers were disembarked at Tenterfield, 20 km south of the Queensland border. Two more trains disgorged their passengers at Tenterfield, swelling the local population by 800 and stretching its amenities to near-breaking. According to the November agreement, the only means by which these travellers could reach Brisbane was by returning to Sydney and proceeding by sea, subject to maritime quarantine measures operated by the Commonwealth. Acting Prime Minister Watt offered the stranded passengers free train travel back to infected Sydney, from where they would need to find accommodation until an infrequent and expensive steamship fare became available.^139^ Despite the representations of the Queensland government, and newspaper columns filled with tales of hardship from displaced Queenslanders living in basic conditions, the Commonwealth refused to countenance a quarantine station on the state border.^140^ The refusal probably reflected Cumpston’s scepticism about the value of land quarantine, as well as the Commonwealth’s reluctance to bear responsibility for an operation that would be run day-to-day by the Queensland authorities; if the quarantine programme failed, the federal government would wear the blame.^141^ Watt wrote to the premiers in early February about the ‘Practical difficulties attending’ quarantine stations:

the risks involved in the event of an outbreak of the epidemic in a hastily established and ill-equipped quarantine station at the border presents such difficulties of tragic results that the Commonwealth does not in any circumstances feel justified in running the risk of establishing border stations, which would necessarily have no proper sanitary or hospital conveniences.^142^

In the absence of Commonwealth support, the Queensland premier announced on 1 February that the border with New South Wales would be subject to strict quarantine procedures.^143^ Quarantine camps were established at Wallangarra, Mugindi, Goondiwindi and Coolangatta, at which travellers were required to spend 7 days.^144^ Queensland also intervened in maritime quarantine processes for both interstate and overseas vessels, much to the irritation of the Commonwealth Director of Quarantine.^145^ ‘Constitutional Crisis’ was the headline of an article in the Brisbane *Telegraph* about the conflicting public health powers of state and federal governments.^146^

Like Victoria, South Australia was laggard in declaring itself infected. A large number of cases of pneumonic influenza had been reported by the medical superintendent of the Adelaide Hospital in late January.^147^ By the time the state gave official notification on 5 February 1919, the disease had been circulating for some time.^148^ South Australia closed its land border to Victoria, until a delegation of Victorian and Commonwealth officials, including Cumpston and Robertson, negotiated an agreement wherein restricted travel could resume.^149^ Tasmania and New South Wales both responded to the South Australian outbreak without waiting for the state’s official notification. On 31 January, Tasmania began controlling the entry of small sailing vessels from South Australia and New South Wales prohibited entry of South Australians.^150^ Cumpston criticised these measures on the basis that South Australia had not officially declared itself to be infected.^151^

## The Trans-Continental Train

Western Australia’s geographical isolation from the rest of continental Australia and its sparse population afforded it greater opportunity of preventing infection altogether.^152^ At the same time, the state’s ambivalence about the Federation found expression in this new threat from the east. The always-opiniated *Sunday Times* warned its readers that the ‘danger that menaces this State resides in the Eastern States’.^153^ Unlike in the other mainland states and Tasmania, it was a train line that became the medium for federal dispute in Western Australia during the pandemic.

Entrepreneurial European Australians long imagined a land connection between the west and the eastern colonies.^154^ A meeting of the Agricultural Society of Western Australia in 1840 considered a South Australian proposal to build a connecting road between the colonies.^155^ The suggestion was ‘fiercely opposed, and ultimately rejected on the grounds of “impracticability and the undesirability of making a road to enable bushrangers from the penal settlements in the East to make raids upon this Colony.”’^156^ Attitudes softened over the years, particularly following the goldrushes of the 1890s, which brought tens of thousands of ‘t′othersiders’ to Western Australia.

The West Australian premier John Forrest became the most forceful advocate for a transcontinental railway, which he argued was ‘necessary to make federation a reality’.^157^ While the project was not made a condition of Federation, it helped convince ambivalent West Australians to join the new Commonwealth. After Forrest’s expectation that the railway would be considered by the first federal parliament was dashed, he threatened to use all his powers to ‘undo’ the Federation should it not be built.^158^ Western Australia passed an enabling bill to construct the railway in 1903, but the Commonwealth dithered and South Australia obfuscated, the latter eyeing a north–south rail link that would position that state, which still included the Northern Territory, as a northern gateway to the nation.^159^ In 1904, the West Australian senator George Pearce asked his colleagues whether they were ‘prepared to make Federation a real thing for Western Australia? Without this railway, “Federation” is a delusion and a sham …’, he said.^160^ Improved national finances and increased anxiety about the vulnerability of the isolated western third of the continent to military aggression led the Fisher Labor government to pass legislation in 1911 authorising the construction of the railway.^161^

The war slowed but did not halt construction of the trans-continental railway, which was completed in October 1917. The committee charged with celebrating the opening of the link, which Forrest had lobbied unsuccessfully to call the ‘Great Western Railway’, appeared more interested in illuminating imperial than national associations.^162^ The railway would be a ‘valuable Empire asset’ and ‘play an important part in Empire politics’, by virtue of enabling Western Australia to ‘take our place on the map as the European gateway to the Commonwealth’, its report noted.^163^ Others more attuned to the national audience described the achievement within the boundaries of the Federation rather than the British empire; ‘East and West are indissolubly joined together by bands of steel’, Forrest announced.^164^ A West Australian trade journal declared that the businessmen of Perth were ‘breathing the Federal spirit’.^165^ The work of the celebration committee came to nought when the official opening ceremony was abandoned due to the ‘grave National conditions’ of late 1917: the tumultuous campaign that was underway for a second vote on conscription, and the dreadful casualty figures from Australian actions at Passchendaele.^166^

When reports of an outbreak of influenza in Victoria began reaching Western Australia in late January 1919, the premier Henry Lefroy was at the Premiers’ Conference in Melbourne, with two of his senior ministers. Lefroy’s absence left the deputy premier and Health Minister, Hal Colebatch, in charge. On 27 January, the day before Victoria’s official notification, a passenger train had departed from Melbourne headed for Western Australia. Presumably, armed with intelligence that the Melbourne situation was worse than health officials were conceding, Colebatch instructed his chief health officer, Everitt Atkinson, to quarantine the train in order to ensure that 7 days would elapse before passengers from Victoria disembarked in Western Australia.^167^ Atkinson notified Cumpston of his intention by telegram on 29 January—despite Cumpston’s latter protestations that he received no warning of state actions—and the following day the Western Australian government detained the train at Parkeston.^168^ Two more trains were held up at Parkeston, a station some distance from Kalgoorlie—itself 500 km east of the state’s capital—after which the Commonwealth cancelled the service.^169^ The West Australian government intended initially to accommodate the quarantined passengers in tents and marquees provided by military authorities. When strong winds blew down the tents, state health officials, in consultation with Commonwealth railways officers, used the train’s dining and sleeping cars to house quarantined staff and passengers.^170^

Watt professed outrage upon news of Colebatch’s decision to ‘seize’ the transcontinental train. He threatened to cancel the service altogether, unless Colebatch released the train. While Colebatch exchanged terse messages with Watt, Henry Lefroy, remained at the Menzies Hotel in Melbourne, along with the state Treasurer, James Gardiner, and the Minister for Works, William George. He was unable to find a passage home due to the cancellation of the inter-continental train and a chronic shortage of shipping. Lefroy was in a difficult position; he was being harried daily by stranded Western Australians demanding he get them home. He described to a colleague the ‘awful situation … four hundred people hammering at my door and I am powerless’.^171^ Lefroy was also being pressured by Watt to bring the recalcitrant Colebatch into line.

Colebatch was resolute in the face of pressure from Watt and Lefroy to resume the train service. In response to Lefroy’s pleadings, the acting premier reminded him of the intense hostility in the west towards the Commonwealth and Victoria.^172^ Colebatch also reminded Lefroy that he acted on advice from his chief medical officer and the medical advisory committee, appointed under the terms of the November agreement, which met twice weekly. Apart from the quarantine power and despite the Commonwealth’s recent encroachments, public health responsibilities rested with state and local governments. While state governments possessed legislative powers, local municipalities played a very significant role in the detection, notification and management of the pandemic. Colebatch wrote to Treasurer Gardiner that: ‘the Government of Western Australia cannot abrogate its sovereign rights to protect the health of its people’.^173^ As their relationship soured, Colebatch let Lefroy know that he was ‘deeply distressed’ by the premier’s refusal to endorse a position that was unanimously held by Cabinet, health authorities and the medical advisory committee and ‘endorsed by all sections of the public’.^174^

## ‘Great Days, Bitter Days’: The November Agreement Abandoned

Watt threatened premiers on 4 February that unless they adhered to the terms of the November agreement and allowed the Commonwealth to police cross-border traffic, it would renounce the pact and ‘revert to the constitutional position it occupied before the agreement was drawn’.^175^ The tone of the telegram was abrupt, accusing New South Wales, Queensland, West Australia and Tasmania of ‘violation’ of the November agreement.^176^ Watt justified his own actions; given the terms of the agreement, the Commonwealth had to await a formal declaration from the Chief Health Officer of Victoria: any other course would have comprised ‘an extensive invasion into the health domain of the States, and the States would have been the first to complain against any such intrusion’.^177^ Despite the seriousness of the outbreak, Watt repeated the Director of Quarantine’s view that the disease spreading in Australia might be different from the one that was circulating in other parts of the world.^178^

Premiers reacted angrily to Watt’s accusation that they had violated the November agreement. Colebatch ‘indignantly repudiate[d] the charge … it seems a monstrous action on the part of the Commonwealth Government to uphold the two States that have violated the agreement, and to prefer an entirely unwarranted charge against those States that have been forced to take action through the violation of the agreement by Victoria and South Australia’.^179^ Holman was equally outraged by Watt’s self-serving account of events and echoed Colebatch’s claim that it was Victoria that had broken the November agreement.

The Commonwealth faced inevitable allegations of bias towards Victoria. The *Sydney Morning Herald* accused the federal government of failing to ‘appreciate public opinion in States other than Victoria’, while the *Brisbane Courier* thought it was not with the states, but with the Commonwealth that ‘the real parochialism lies’.^180^ Implicit in the prime minister’s actions, Colebatch claimed, was ‘the suggestion that now Victoria has become infected there is not the same need for precautionary measures throughout Australia as existed previously’.^181^

What the Western Australian public are asking themselves is whether if in this State there existed the condition of things now existing in Victoria, Mr Watt would be so keen upon insisting on free intercourse with Western Australia as he is now upon our opening our back door to the disease from Adelaide and Melbourne.^182^

While the federal government maintained such an attitude, Colebatch said, ‘co-operation between the States and the Commonwealth is impossible’.^183^ The *West Australian* newspaper looked ‘in vain to find among Mr Watt’s communications any expression of reproach to the Victorian and South Australian departments because of their failure to notify the presence of the disease in their respective States’.^184^ The *West* was unsure of the Commonwealth’s motivation: ‘Whether it is because the Federal Government is subject to Victorian influences, or that it attaches a supreme importance to its dignity and constitutional position, is hard to determine’.^185^ The *Sunday Times* labelled William Watt a ‘jumped-up jack-in-office’ and ‘tin-pot tyrant’.^186^ ‘How long will the West Australian people suffer the intolerable insolence of these elected persons in the East?’, the paper asked.^187^

Many suspected that geographical proximity played a role in the Commonwealth’s inexplicable failure to chastise Victoria for its silence during those crucial weeks in early January. Watt himself had Victorian connections that left him vulnerable to accusations of bias and cronyism. He was born near Kyneton, north-west of Melbourne, in 1871 and first elected to the Victorian parliament as a progressive liberal in 1897. Watt served as Victorian premier between 1912 and 1914, before switching to federal politics after defeating Labor candidate John Curtin for the seat of Balaclava at the 1914 election. He knew Lawson and many of his ministers and officials personally. Suspicions of partiality extended beyond the Victorian roots and connections of the acting prime minister. The historian Paul Strangio has documented the resentment of New South Wales, in particular, about the Commonwealth’s Victorian base during the decade after Federation and argues there was good reason for it—Victorian members were greatly advantaged by their proximity to parliament and able to attend far more regularly than their interstate counterparts. They benefited also from their easy access to government departments and officials.^188^ It is reasonable to assume that this privilege continued until the parliament moved to Canberra in 1927, when resentment could coalesce around a city unencumbered by state as well as federal allegiances.

Watt rebuffed claims that he was biased towards Victoria, but the accusation stuck. Feeling against the Commonwealth was most intense in Western Australia, where civic institutions, organised labour and newspaper editorials rallied behind Colebatch’s uncompromising stance.^189^ Existing resentment of the Federation and the Commonwealth was intensified by the Spanish flu episode. The *Fremantle Times* estimated that 95 per cent of West Australians would support secession if a vote were held immediately.^190^ The *West Australian* thought the quarrel over the trans-Australian railway was ‘another instance, added to not a few prior examples, of the doubtful wisdom of entrusting too much power to a central Government’.^191^ There was never serious momentum for separation, but the occasion gave those who were permanently aggrieved by the federal settlement another opportunity to rehearse their claims. A citizen’s meeting was called in the Perth Town Hall on 10 March 1919 to discuss secession. The main speakers, a state and a federal parliamentarian, both declared themselves federalists, then listed their grievances against the Federation—the Commonwealth’s extravagance, the failure to develop Western Australian industry, the lack of support for development of the north-west of the state—but pulled up short of advocating secession.^192^ If secession proved impossible, the *Fremantle Times* called instead for reform of the Senate, which had ceased to function as a voice for the states and become, like the House of Representatives, a province for party allegiance.^193^

The chief source of discontent in the West remained the belief that the tariff imposed on imported goods had disadvantaged Western Australian primary industries by increasing the cost of machinery, and smothered nascent manufacturing industries, which were unable to match their eastern states competition. Western Australia had suffered financially, while the Commonwealth spent extravagantly and a ‘gang of Eastern commercial vultures’ aided by their pawns in parliament, profited enormously.^194^ That the Commonwealth defended Victoria’s actions in early 1919 and blamed the other states unjustly, only exacerbated the impression among Western Australians that they were the poor relations of the Federation. In her history of the pandemic in Western Australia, Bev Blackwell argued that the state’s ability to resist the disease for several months: ‘reaffirmed the proud belief of West Australians that they were different from the eastern states’.^195^

Though *popular* feeling did not run so deeply against the Commonwealth in New South Wales, there was immense ‘official hostility’ between the two spheres.^196^ Cumpston later recalled a meeting with the New South Wales Cabinet at which he was ‘attacked by all the Ministers’ and eventually walked out.^197^ He claimed that Holman, who had not been present, rang him the following day to apologise for the rudeness of his colleagues.^198^ ‘Great days, bitter days, such as I should not like to live through again’, he recalled.^199^

Faced with the defiance of all states except Victoria, the Commonwealth formally renounced the November agreement on 6 February 1919. It declared its intention to persist with the overseas quarantine restrictions in operation since October 1918 but issued new regulations under the *Quarantine Act* in relation to interstate sea quarantine.^200^ Cumpston was now convinced that the influenza had an incubation period of 48 h and 72 at most, thus justifying the reduction of quarantine from 7 to 4 days.^201^ Queensland and Western Australia found these new regulations insufficiently rigorous and continued to make their own arrangements. The Commonwealth allowed the West Australian government to utilise Garden Island as a quarantine station for passengers to serve out the remaining 3 days after release by Commonwealth authorities.^202^ Cumpston was typically self-serving about the collapse of the agreement, telling Watt that the states, ‘with the exception of Victoria and South Australia’ were interpreting it as they see fit or ignoring it altogether; an interpretation with which Watt concurred, at least publicly.^203^

## Compromise and Retribution

In later publications, Cumpston claimed vindication for his scepticism about the value of land quarantine, claiming that efforts at border quarantine proved ‘farcical’ and allowed the virus to spread throughout the country.^204^ According to the biography of Cumpston written by his daughter, Margaret Spencer:

one after another the State Governments admitted the futility of all these restrictive measures, and invited the Commonwealth to take control. There was thus no question of over-riding Commonwealth action. Cumpston initiated a simple system of control over rail traffic, medical inspection before departure and surveillance at destination, but made no attempt to control other land traffic. This system was established to enable State Governments to disengage without loss of prestige, but that it was merely formal and practically valueless was recognized from the beginning.^205^

The evidence suggests a different version of events. The federal government worked hard to repair connections by land and sea and was prepared to compromise to that end. Once South Australia was declared ‘clean’ on 13 March, negotiations for the resumption of the Trans-Australian Railway proceeded in earnest.^206^ Lefroy and Colebatch refused to compromise on their strict quarantine conditions for the train, which would be enforced by Commonwealth quarantine officers.^207^ The first batch of westward-bound passengers went into quarantine at Karonie, 100 km east of Kalgoorlie, on 10 April 1919. The camp was equipped by the Commonwealth Works Department with tents, sanitary arrangements and a store selling tobacco and ‘other luxuries’.^208^ Catering was provided by the Commonwealth at a cost of 10 shillings per day.

South Australia allowed Commonwealth control of its borders in early February while New South Wales transferred responsibility in mid-April 1919. Queensland, Western Australia and Tasmania retained their 7-day quarantine measures on both overseas and domestic shipping, despite Commonwealth threats to reduce their shares of tonnage in coastal shipping. Tasmanians interpreted Watt’s threat as a sign that he prioritised mainland commercial interests over the health of Tasmanians.^209^ When the Commonwealth disembarked returned soldiers at Lytton quarantine station near Brisbane after serving only 4 days’ quarantine, the Queensland government sought an injunction from the High Court. Justice Duffy delayed his decision and in the meantime an agreement was reached in late May whereby Queensland ceded to Commonwealth quarantine controls.^210^

Western Australia was closed off by land and increasingly by sea, due to industrial disputes and the actions of the federal government. It faced chronic shortages of coal, timber, butter and other food supplies. There were cries of retribution when the Commonwealth increased freight costs and then cancelled ships bringing coal to Western Australia from Newcastle after lumpers refused to allow non-unionists to unload the *Dimboola*.^211^ The state Industries Minister wrote to Colebatch: ‘Either the Commonwealth Authorities are densely stupid, or else this rise in freight costs of 7/6 per ton is by way of retaliation to Western Australia’.^212^ The Commonwealth also required overseas ships to quarantine for 7 days at Fremantle, despite it being a clean port. Robinson told Gardiner that the onerous quarantine regulations were ‘childish and [amount] to retaliation pure and simple’.^213^ Colebatch condemned the ‘unwarrantable aggression’ of the Commonwealth, and the federal member for Perth asked Watt whether he wanted to provoke the ‘West into open rebellion against [the] Federal government’.^214^ Colebatch and others pointed out that the cancelled colliers would become available for the Victorian trade, in time to prevent the loss of as many as 100,000 jobs in industry due to the coal shortage.^215^

Colebatch, who had replaced Lefroy as premier on 15 April, repeatedly asked the Commonwealth to settle the dispute on the Fremantle wharves between the federal labour force and the union lumpers, but Watt refused to intervene.^216^ With supplies on the *Dimboola* desperately needed to combat shortages, Colebatch proceeded to Fremantle on the morning of 4 May 1919, where the national workers were hoping to unload the ship without union knowledge. The unionists were waiting, however, and a showdown between police and lumpers—the ‘Battle of the Barricades’—led to the death of one man and helped make Colebatch’s the shortest premiership in the history of the state. He resigned on 13 May 1919, having been premier for exactly 1 month.

Despite Cumpston’s criticism of land quarantine measures, they did delay the spread of the virus. Queensland held off the virus until the end of April and Western Australia until early June 1919. Tasmania remained ‘clean’ until mid-August.^217^ Measures on land had a similar effect to Cumpston’s maritime quarantine; they hindered the spread of the virus to new populations and probably decreased its virulence. The mortality rate in New South Wales was 304 per 100,000 and in Victoria it was 243 per 100,000, though the latter figure is likely a significant underestimate. In Queensland, the rate was 156, Western Australia 167, and Tasmania 114 per 100,000.^218^

After federal parliament rose for the Christmas break in December 1918, it did not return until June the following year because of the pandemic. The acrimony within the Federation was evident in Watt’s address to the House of Representatives on 25 June 1919. He spoke about the abrogation of the November agreement by the states, ‘in defiance of constitutional rights’, which had created ‘lamentable disorganization of the shipping services, occasioning serious shortages of food supplies and fuel in many parts of the Commonwealth, and grave delays in the debarkation of our returning soldiers’.^219^ Cumpston, as we have seen, was even more brazen. In numerous publications over subsequent decades, he cast himself as the diligent bureaucrat urging Victorian officials to declare the presence of pneumonic influenza, but unable to directly intervene for fear of breaching constitutional propriety. In truth, Cumpston’s reluctance to entertain the prospect that his maritime quarantine barrier had been breached, and his scepticism about land quarantine, led him to obfuscate. If not for the collusion between Cumpston and Victorian officials to delay notification in mid-January 1919, the influenza would not have spread so quickly in Melbourne and crossed into New South Wales as soon as it did. Cumpston’s actions and his subsequent misrepresentations of them undermine the great achievement of his maritime quarantine.

## Conclusion

The maritime quarantine did not function during the Spanish influenza pandemic as a bio-political border that reinforced a sense of Australian nationhood. Even while Cumpston’s maritime perimeter remained intact, states protested the actions of the federal government in enforcing its quarantine power. Once the disease broke through the maritime border, colonial loyalties asserted themselves even more forcefully. Rather than drawing a metaphorical boundary around the racialised Australian nation, quarantine divided the Commonwealth along its colonial borders. Tasmania felt more neglected, New South Wales and Queensland more resentful towards Victoria and the Commonwealth, and Western Australia more isolated and aggrieved. All resented Victoria, which they believed had colluded with the Commonwealth.

The experience of the Spanish influenza in Australia demonstrates that, while public health practices frequently act as centripetal forces, which enhance the authority of the central government, they can also act centrifugally, exacerbating divisions and intensifying subnational loyalties. This suggests a need to think more carefully about how governmental authority functions in federal nation-states during public health crises, not least because a similar pattern is observable during the COVID-19 pandemic. In the Australian setting, state governments have been more prominent and forceful than the Commonwealth in their management of the virus.^220^ The Commonwealth has foregone its constitutional jurisdiction over quarantine, leaving the states to run hotel quarantine programmes for people returning from overseas. Data regarding infection numbers and testing processes are collected at the state level and often communicated by premiers themselves. The Victorian premier, Daniel Andrews, conducted live press briefings for 120 days consecutively during that state’s mid-2020 outbreak.^221^ The Commonwealth is widely perceived to have mismanaged the COVID-19 vaccination programme, which has been hampered by delays and very widespread reluctance among a population that is both complacent about the risk of infection and concerned about side-effects from the vaccination.^222^ In contrast to both the criticisms of federalism expressed at the opening of this article, and bio-political readings of public health practices, the case of the Spanish influenza in Australia suggests that public health emergencies narrow the communal parameters within which citizens feel secure as well as the bureaucratic capacity to respond effectively. The evidence of COVID-19 is yet to be weighed.

## Footnotes

1 Niall Ferguson, *Doom: The Politics of Catastrophe* (London: Penguin Press, 2021); Covid Performance Index: Deconstructing Pandemic Responses, Lowy Institute, https://interactives.lowyinstitute.org/features/covid-performance/; accessed 17 May 2021.

2 Tahla Burki, ‘China’s Successful Control of COVID’, *Lancet*, 2020, 20, 240–41; Covid Performance Index, Lowy Institute.

3 Nicole Huberfeld, Sarah H. Gordon and David K. Jones, ‘Federalism Complicates the Response to the COVID-19 Health and Economic Crisis: What Can Be Done?’, *Journal of Health Politics, Policy and Law*, 2020, 45, 951.

4
*Ibid*., 952.

5 Bill Bowtell, *Unmasked: The Politics of Pandemics* (Melbourne: Monash University Press, 2021), 75.

6
*Ibid*., vi.

7 A similar, if not quite so dramatic, phenomenon occurred in Queensland, where Labor Premier Annastacia Palaszczuk, who had also resisted pressure to reopen the state border, won an emphatic election victory in October 2020.

8 Dorothy Porter, *Health, Civilization and the State: A History of Public Health from Ancient to Modern Times* (London: Routledge, 1998), 3–11; Frank M. Snowden, *Epidemics and Society: From the Black Death to the Present* (New Haven: Yale University Press, 2020), 69–82.

9 Snowden, *Epidemics and Society*, 82.

10 For example, Michel Foucault, ‘The Politics of Health in the Eighteenth Century’, in Colin Gordon ed., *Power/Knowledge: Selected Interviews and Other Writings, 1972-1977* (New York: Pantheon, 1980), 166–86; Michel Foucault, ‘The Birth of Biopolitics’; ‘Security, Territory and Population’; ‘On the Government of Living’, in Paul Rabinow, ed., *The Essential Works of Foucault, 1954-1984*, vol. 1*: Ethics: Subjectivity and Truth* (New York: New Press, 1997), 51–86; Robin Bunton and Alan Petersen, eds, *Foucault, Health and Medicine* (London: Routledge, 1997).

11 Michel Foucault, *The Will to Knowledge: The History of Sexuality*, vol. 1, Robert Hurley (trans) (London: Penguin, 1998 [1976]), 139.

12
*Ibid*., 140–41.

13 Alison Bashford, ‘Quarantine and the Imagining of the Australian Nation’, *Health*, 1998, *2*, 390.

14 Warwick Anderson, *The Cultivation of Whiteness: Science, Health, and Racial Destiny in Australia*, (Melbourne: Melbourne University Press, 2002). See also, Christopher Mayes, ‘White Medicine, White Ethics: On the Historical Formation of Racism in Australian Healthcare’, *Journal of Australian Studies*, 2020, 44, 287–302; Karen Agutter and Catherine Kevin, ‘Lost in Translation: Managing Medicalised Motherhood in Post-World War Two Australian Migrant Accommodation Centres’, *Women’s History Review*, 2018, 27, 1065–85; F. B. Smith, ‘Medical Inspection of State Schoolchildren in Australia, c. 1905-14’, *Health and History*, 2008, 10, 5–20; Lisa Featherstone, ‘The Value of an Infant: The Rise of Paediatrics in Australia, 1880-1910’, *Health and History*, 2008, 10, 110–133.

15 Warwick Anderson, ‘Coolie Therapeutics: Labor, Race, and Medical Science in Tropical Australia’, *International Labor and Working-Class History*, 2017, 91, 46–58.

16 Mayes, ‘White Medicine, White Ethics’.

17 Humphrey McQueen, ‘The “Spanish” Influenza Pandemic in Australia—1918-19’, in *Social Policy in Australia: Some Perspectives, 1901-1975* (Sydney: Cassel, 1976), 142; Michael Roe, ‘The Establishment of the Australian Department of Health: Its Background and Significance’, *Australian Historical Studies*, 1976, 17, 190.

18 Roe, ‘The Establishment of the Australian Department of Health’, 191.

19 Alison Bashford, ‘Insanity and Immigration Restriction’, in Catherine Cox and Hilary Marland, eds., *Migration, Health, and Ethnicity in the Modern World* (Basingstoke: Palgrave, 2013), 14; Alison Bashford, ‘Internationalism, Cosmopolitanism, and Eugenics’, 158–60; Stephen Garton, ‘Eugenics in Australia and New Zealand’, 244; and Linda Bryder, ‘The History of Medicine in Australia and New Zealand’, in Mark Jackson, ed., *The History of Medicine* (Oxford: Oxford University Press, 2011), 313; Jennifer S. Kain, ‘Standardising Defence Lines: William Perrin Norris, Eugenics and Australian Border Control’, *Social History of Medicine*, 2018, 33, 843–59.

20 Alison Bashford, *Imperial Hygiene: A Critical History of Colonialism, Nationalism and Public Health* (Basingstoke: Hants Palgrave, 2004), 123.

21
*Ibid*., 124.

22 Alison Bashford, ‘At the Border: Contagion, Immigration, Nation’, *Australian Historical Studies*, 2002, 33, 350; also, Bashford, ‘Quarantine and the Imagining of the Australian Nation’, 387–402. Krista Maglen qualifies Bashford’s argument somewhat by showing that quarantine practices were more likely to isolate ships from Europe and America than ships from Asia; Krista Maglen, ‘A World Apart: Geography, Australian Quarantine, and the Mother Country’, *Journal of the History of Medicine and Allied Sciences*, 2005, 60, 196–217. Bashford herself has muted her argument about the role of quarantine in shaping racialised nationalism in Alison Bashford and Peter Hobbins, ‘Rewriting Quarantine: Pacific History at Australia’s Edge’, *Australian Historical Studies*, 2015, 46, 392–409.

23 For example, Robyn Arrowsmith, *A Danger Greater than War: NSW and the 1918-1919 Influenza Pandemic* (Canberra: Australian Homeland Security Research Group, 2007); Bev Blackwell, *Western Isolation: The Perth Experience of the 1918-1919 Influenza Pandemic* (Canberra: Australian Homeland Security Centre, 2007); Pauline Curby, *Memories of the 1919 Influenza Pandemic* (Ryde: Ryde City Council, 1998); Peter Curson, *Deadly Encounters: How Infectious Disease Helped Shape Australia* (Bury St Edmonds: Arena Books, 2015); Patrick George Hodgson, ‘Flu, Society and the State: The Political, Social and Economic Implications of the 1918-1920 Influenza Pandemic in Queensland’ (unpublished PhD thesis, James Cook University, 2017); Jean Duncan Foley, *In Quarantine: A History of Sydney’s Quarantine Station, 1828–1984* (Kenthurst: Kangaroo Press, 1995); Peter Hobbins, Ursula K. Frederick and Anne Clarke, *Stories from the Sandstone: Quarantine Inscriptions from Australia’s Immigrant Past* (Crows Nest: Arbon Publishing, 2016); Jeremy Youde, ‘Covering the Cough? Memory, Remembrance, and Influenza Amnesia’, *Australian Journal of Politics and History*, 2017, 63, 357–68. Ian W. Shaw’s recent trade publication, *Pandemic: The Spanish Flu in Australia, 1918-1920* (Sydney: Woodslane Press, 2020), takes a national view of the pandemic.

24 See, for example, Arrowsmith, *A Danger Greater than War*, 73; Anthea Hyslop, ‘Insidious Immigrant: Spanish Influenza and Border Quarantine in Australia, 1919’, in Suzanne Parry, ed., *From Migration to Mining: Medicine and Health in Australian History* (Darwin: Northern Territory University Press, 1998), 213; James A. Gillespie, *The Price of Health: Australian Governments and Medical Politics, 1910-1960* (Cambridge: Cambridge University Press, 1991), 37.

25 ‘Anzac’ is an acronym that stands for Australian and New Zealand Army Corps. For a detailed history of the Anzac legend, see Carolyn Holbrook, *Anzac: The Unauthorised Biography* (Sydney: New South, 2014).

26 See Holbrook, *Anzac: The Unauthorised Biography*.

27 C. E. W. Bean, *The Story of Anzac: From 4 May 1915 to the Evacuation of the Gallipoli Peninsula*, vol. II, *The Official History of Australia in the War of 1914—1918*, new edn (Brisbane: QUP, 1981 [1924]), 910; Humphrey McQueen, ‘The “Spanish” Influenza Pandemic in Australia’, 143.

28 Porter, *Health, Civilization and the State*, 110.

29 Bashford and Hobbins, ‘Rewriting Quarantine’, 392.

30 Maglen, ‘A World Apart’, 196–217; Snowden, *Epidemics and Society*, 184–203.

31 British *Hansard*, 12, 1825, 993, quoted in J. C. McDonald, ‘The History of Quarantine in Britain in the Nineteenth Century’, *Bulletin of the History of Medicine*, 1951, 25, 26, quoted in Maglen, ‘A World Apart’, 197.

32 Maglen, ‘A World Apart’, 199.

33 Hodgson, ‘Flu, Society and the State’, 57.

34
*Ibid*.

35
*Ibid*.

36 Maglen, ‘A World Apart’, 214.

37 J. H. L. Cumpston, *The Health of the People: A Study in Federalism* (Canberra: Roebuck, 1978), 9.

38 Bashford, *Imperial Hygiene*, 120.

39 Charles K. Mackellar, ‘Federal Quarantine’, paper read to the Royal Society of New South Wales, July 1883, quoted in Cumpston, *The Health of the People*, 10.

40 Bashford, *Imperial Hygiene*, 20.

41 This was at the 1897–98 federal convention in Adelaide, Milton J. Lewis, *The People’s Health: Public Health in Australia, 1788-1950* (Westport: Praeger, 2003), 180.

42 Cumpston, *The Health of the People*, 18.

43
*Ibid*., 19; Lewis, *The People’s Health*, 80.

44
*Medical Journal of Australia*, 5 February 1955, 191.

45
*Ibid*.

46 Cumpston, *The Health of the People*, 16.

47
*Ibid.*, 29.

48 Roe, ‘The Establishment of the Australian Department of Health’, 184.

49
*Ibid*., 184–85.

50 Hon. James Stopford, Home Secretary of Queensland, speech in Queensland Parliament on passage of *Queensland Hospital Act*, 1923, Queensland *Hansard*, no. 17, 3rd session of 23rd state parliament, 987–92, quoted in Francesca Beddie, *Putting Life into Years: The Commonwealth’s Role in Australia’s Health Since 1901* (Canberra: Commonwealth Department of Health, 2001), 9.

51 Stopford, quoted in Beddie, *Putting Life into Years*, 9.

52 The *Medical Journal of Australia* was run independently of the British Medical Association of Australia, but in practice their views were closely aligned, Claudia Thame, ‘Health and the State: The Development of Collective Responsibility for Health Care in Australia in the First Half of the Twentieth Century’ (unpublished PhD thesis, Australian National University, 1974), vi.

53
*Medical Journal of Australia*, 30 November 1918, 445.

54
*Ibid*.

55 Gillespie, *The Price of Health*, 31–57; Roe, ‘The Establishment of the Australian Department of Health’, 176–92.

56 Roe, ‘The Establishment of the Australian Department of Health’, 191.

57 C. E. W. Bean, *In Your Hands, Australians*, 2nd edn (London: Cassell, 1919), 36.

58 Claudia Thame, ‘Health and the State’, 25–31.

59 See J. H. L. Cumpston, ‘The War and Public Health’, in Richard J. A. Berry and Archibald T. Strong eds, *Public Lectures on the War* (Melbourne: George Robertson and Co, 1916), 193, 195.

60 Cumpston, *The Health of the People*, 30–33.

61 Victor Heiser, *A Doctor’s Odyssey: Adventures in Forty-Five Countries* (London: Jonathan Cape, 1937), 360–61.

62
*Ibid*., 361.

63 John S. Oxford and Douglas Gill, ‘A Possible European Origin of the Spanish Influenza and the First Attempts to Reduce Mortality to Combat Superinfecting Bacteria: An Opinion from a Virologist and a Military Historian’, *Human Vaccines & Immunotherapeutics*, 2019, 15.

64
*Medical Journal of Australia*, 23 November 1918, 437.

65 Adelaide *Register*, 25 November 1918, 6.

66 Melbourne *Herald*, 15 November 1918, 1.

67 Cumpston, *The Health of the People*, 35.

68 NSW *Hansard*, 25 November 1918, 2934.

69
*Ibid*., 2938.

70
*Ibid*., 2941.

71
*Medical Journal of Australia*, 7 December 1918, 472.

72
*Ibid*.

73 Blackwell, *Western Isolation*, 5.

74
*West Australian*, 6 December 1918, 6.

75
*West Australian*, 16 December 1918, 6.

76 Brisbane *Courier*, 9 December 1918, 7.

77
*Medical Journal of Australia*, 11 January 1919, 39.

78
*Ibid.*, 7 December 1918, 472.

79 Copy of telegram from Premier [signed George Fuller] to Massy Greene, 23 November 1918, NAA: 1919/482 PART 2; NSW *Hansard*, 25 November 1918, 2933.

80 The disease was officially given this title at the November 1918 conference of the Commonwealth and state governments, ‘Commonwealth and States of Australia Influenza Conference 1918’, NAA: 1919/482 PART 2.

81 ‘Influenza Conference, 1918, Resolutions’, ‘Commonwealth and States of Australia Influenza Conference 1918’, NAA: 1919/482 PART 2.

82 Cumpston, *The Health of the People*, 36; Margaret Spencer, *J.H.L. Cumpston, 1880-1954: A Biography* (Tenterfield NSW: self-published, 1987), 165.

83 Blackwell, *Western Isolation*, 65; J. H. L. Cumpston, *Influenza and Maritime Quarantine in Australia* (Melbourne: Commonwealth of Australia, 1919), 65.

84 ‘Commonwealth and States of Australia Influenza Conference 1918’, NAA: 1919/482 PART 2.

85
*Ibid*.

86 Cumpston, *The Health of the People*, 36.

87 For details about W. A. Watt, see John Anderson and Geoffrey Serle, ‘Watt, William Alexander (1871–1946)’, Australian Dictionary of Biography, National Centre of Biography, Australian National University, http://adb.anu.edu.au/biography/watt-william-alexander-9011/text15869, published first in hardcopy 1990, accessed online 24 July 2020.

88 Memorandum from Cumpston to Comptroller-General of Trade and Customs, 22 January 1919, NAA: A1928, 443/11A—‘Health—Department of. Conferences: Federal & State Ministers 1919—Co-ordination of C/wealth and State Powers with respect to Quarantine and other diseases’.

89
*Ibid*.

90
*Ibid*.

91
*Ibid*.

92 Memorandum from Cumpston to Massy-Greene, 8 January 1919, NAA: A1928, 443/11A.

93 It is unclear precisely when the disease appeared in Melbourne. The *Medical Journal of Australia* claimed the first cases were diagnosed in Melbourne on 9 January 1919, *Medical Journal of Australia*, 23 August 1919, 161. Cumpston also identifies 9 January 1919 as the date of the ‘first known case’, *The Health of the People*, 36. A senior honorary physician at St Vincent’s Hospital in Melbourne, Alex Lewers, described a trio of suspicious cases in November 1918, before a spate of cases in ‘early January’ 1919, *Medical Journal of Australia*, 30 August 1919, 168. Lewers contacted the chief health officer of Victoria, Edward Robertson, who told Lewers that no cases had been reported, *Medical Journal of Australia*, 30 August 1919, 168. Cases of ‘influenzal pneumonia’ had appeared in Melbourne on 19 January, *Medical Journal of Australia*, 30 August 1919, 168.

94 Melbourne *Argus*, 23 January 1919, 7.

95
*Ibid*.

96 Anthea Hyslop, ‘A Question of Identity: J.H.L. Cumpston and Spanish Influenza, 1918-1919’, *Australian Cultural History*, 1997–98, 16, 67.

97 Spencer, *J.H.L. Cumpston*, 166.

98 Cumpston, *The Health of the People*, 36.

99
*Ibid*.

100 Sydney *Sun*, 23 January 1919, 7.

101
*Age*, 24 January 1919, 7.

102 Sydney *Sun*, 23 January 1919, 7.

103 Sydney *Daily Telegraph*, 24 January 1919, 5.

104
*Age*, 24 January 1919, 7.

105
*Ibid*.

106
*Ibid*.

107
*Ibid*.

108
*Ibid*.

109
*Ibid*.

110
*Ibid*.

111 Spencer, *J.H.L. Cumpston*, 166; Adelaide *Register*, 25 January 1919, 9.

112 Spencer, *J.H.L. Cumpston*, 166.

113 Hobart *Mercury*, 25 January 1919, 7. Cumpston claims in *The Health of the People* that the presence of the virus in Melbourne was ‘definitely confirmed’ for the first time at the Premiers’ Conference on 23 January and that the meeting was abandoned, Cumpston, *The Health of the People*, 36.

114 Telegram from Henry Lefroy to Hal Colebatch, 27 January 1919, SROWA: S36 Cons1496 1919/0001 Premiers & Ministers Conference Melbourne Jan 1919.

115 Sydney *Daily Telegraph*, 24 January 1919, 5.

116 ‘Report of the Resolutions, Proceedings, and Debates of the Conference of Commonwealth and State Ministers Held at Melbourne, 22–27 January 1919, Commonwealth Govt, u.d. [1919?], pp. 75-7, in NAA: A1928, 443/11A’.

117
*Ibid*.

118
*Ballarat Star*, 27 January 1919, 3.

119 Reports of Watt’s closing speech to the conference, for example, Hobart *Mercury*, 28 January 1919, 4.

120
*Medical Journal of Australia*, 1 February 1919, 92.

121
*Sunday Times*, 26 January 1919, 1.

122 ‘Influenza Epidemic, 1918’, NAA: A6006, 1919/2/3.

123
*Medical Journal of Australia*, 1 February 1919, 92.

124 Adelaide *Register*, 29 January 1919, 8.

125
*West Australian*, 24 February 1919, 4.

126
*Ibid*.

127 Briefing note by Cumpston?, n.d. [early February 1919?], NAA: A6006 1919/2/3 – Influenza Epidemic 1918.

128 Cumpston, *The Health of the People*, 36.

129
*Mildura Cultivator*, 1 February 1919, 7.

130 ‘Influenza Epidemic, 1918’, NAA: A6006, 1919/2/3; *Medical Journal of Australia*, 26 April 1919, 331; Cumpston, *The Health of the People*, 38.

131
*Sydney Morning Herald*, 4 February 1919, 8.

132
*Ballarat Star*, 4 February 1919, 1.

133
*Sydney Morning Herald*, 12 February 1919, 10; telegram from W. Holman to W. A. Watt, 5 February 1919, NAA: A2, 1919/1302—Influenza Epidemic Commonwealth Regulations; *Sydney Morning Herald*, 12 February 1919, 10.

134 Telegram from W. Holman to W. A. Watt, 5 February 1919, NAA: A2, 1919/1302.

135 Cumpston, *The Health of the People*, 36.

136 Hodgson, ‘Flu, Society and the State’, 86.

137
*Ibid.*

138 Cumpston, *The Health of the People*, 36.

139 Hodgson, ‘Flu, Society and the State’, 90.

140
*Ibid*., 91.

141 Blackwell, *Western Isolation*, 97.

142 Extract from letter sent from Acting Prime Minister W. A. Watt to premiers of all states, 6 February 1919, NAA: A2, 1919/482 PART 1.

143 ‘Influenza Epidemic, 1918’, NAA: A6006, 1919/2/3.

144 Cumpston, *The Health of the People*, 36.

145
*Ibid*.

146 Brisbane *Telegraph*, 4 February 1919, 5.

147 Despite the fact that a large number of cases of pneumonic influenza were reported by the medical superintendent of the Adelaide Hospital in late January, South Australia did not declare itself an infected state until 5 February 1919, Telegram from H. B. Lefroy to W. A. Watt, 6 February 1919, NAA: A2, 1919/1302, *West Australian*, 5 February 1919, 7; *West Australian*, 5 February 1919, 7.

148 Telegram from H. B. Lefroy to W. A. Watt, 6 February 1919, NAA: A2, 1919/1302—Influenza Epidemic Commonwealth Regulations.

149
*Horsham Times*, 8 April 1919, 5.

150 ‘Influenza Epidemic, 1918’, NAA: A6006.

151
*Ibid*.

152
*Sunday Times*, 17 August 1919, 4.

153
*Ibid.*, 13 July 1919, 4.

154 David Burke, *Road**through the Wilderness* (Sydney: New South Wales University Press, 1991), xx–xxi.

155
*Report of the Committee Appointed to Formulate a Scheme for Holding an Exposition to Celebrate the Opening of the Trans-Australian Railway*, Perth, 1917, State Library of Western Australia, n.p.

156
*Ibid*. The Governor General did appear for a brief opening ceremony in Perth on 16 November 1917, Burke, *Road through the Wilderness*, 249.

157
*Mercury*, 24 January 1901, 3.

158 Burke, *Road**through the Wilderness*, 53.

159
*Ibid*., 58.

160
*Hansard*, 14 December 1904, 9.

161 Burke, *Road**through the Wilderness*, 73.

162
*Ibid*., 246.

163
*Report of the Committee Appointed to Formulate a Scheme for Holding an Exposition to Celebrate the Opening of the Trans-Australian Railway*, Perth, 1917, State Library of Western Australia, n.p.

164 Burke, *Road**through the Wilderness*, 247.

165
*Land and Transport Journal*, Burke, *Road**through the Wilderness*, 248.

166 Burke, *Road**through the Wilderness*, 247.

167
*West Australian*, 11 February 1919, 5.

168 Telegram from Hal Colebatch to Henry Lefroy, 31 January 1919, SROWA: S36 Cons1496 1919/0001 Premiers & Ministers Conference Melbourne Jan 1919; ‘Influenza Epidemic, 1918’, NAA: A6006, 1919/2/3; Spencer, *J.**H.**L. Cumpston*, 167.

169 Letter from W. A. Watt to Henry Lefroy, 4 February 1919, Telegram from Hal Colebatch to Henry Lefroy, 31 January 1919, SROWA: S36 Cons1496 1919/0001 Premiers & Ministers Conference Melbourne Jan 1919.

170 R. C. Everitt Atkinson, *Report for the Two Years Ending 31^st^ December 1919 upon Medical, Health, Factories and Early Closing Departments* (Perth: West Australian Government, 1920), 10; Telegram from Hal Colebatch to Henry Lefroy, 30 January 1919, SROWA: S36 Cons1496 1919/0001 Premiers & Ministers Conference Melbourne Jan 1919.

171 Telegram from Lefroy to Baxter (Agricultural Department, WA), 19 February 1919, SROWA: S36 Cons1496 1919/0001 Premiers & Ministers Conference Melbourne Jan 1919.

172 Telegram from H. P. Colebatch to Henry Lefroy, 8 February 1919, SROWA: S36 Cons1496 1919/0001.

173 Telegram from Colebatch to Gardiner, 3 March 1919, SROWA, S36 Cons1496 1919/0033 Influenza Epidemic in Eastern States.

174 For example, Telegram from Colebatch to Lefroy, 12 February 1919, Telegram from Colebatch to George, 15 February 1919, SROWA: S36 Cons1496 1919/0050 Influenza – uniformity of action re stranded citizens.

175 Telegram from W. A. Watt to state premiers, 3 February 1919, NAA: A2, 1919/1302—Influenza Epidemic Commonwealth Regulations.

176 Telegram from W. A. Watt to state premiers, 3 February 1919, NAA: A2, 1919/1302.

177 ‘Quarantine’, *Mercury*, 12 February 1919, 5.

178
*Ibid*.

179 Telegram from H. P. Colebatch to W. A. Watt, 4 February 1919, NAA: A2, 1919/1302; *West Australian*, 5 February 1919, 7.

180
*Sydney Morning Herald*, 29 January 1919, n.p., quoted in Hyslop, ‘Insidious Immigrant’; *Brisbane Courier*, 7 February 1919, n.p., quoted in Suzanne Parry, ed., *Migration to**Mining: Collected Papers of the Fifth Biennial Conference of the Australian Society of the History of Medicine* (Darwin: Northern Territory University Press, Darwin, 1998), 209.

181
*Daily News*, 12 February 1919, 6.

182
*Western Mail*, 14 February 1919, 27.

183
*Daily News*, 12 February 1919, 6.

184
*West Australian*, 6 February 1919, 4.

185
*Ibid*.

186
*Sunday Times*, 9 February 1919, 7.

187
*Ibid*.

188 Paul Strangio, ‘“Dished Like a Dinner” by the Victorians? The 1899 Seat of Government Compact and Its Repercussions in the Early Commonwealth’, *Australian Journal of Politics and History*, 2019, 65, 46–49.

189 For example, Letter from Albert E. King, Perth Chamber of Commerce to Senator Hon. Patrick J. Lynch, 25 April 1919, NAA: CP103/11—[Influenza. Miscellaneous correspondence]; Telegram from Secretary Labor Party, Kalgoorlie to Acting Prime Minister Watt, 8 April 1919, NAA: CP103/11 415—Influenza Epidemic. Transcontinental Railways Miscellaneous; *West Australian*, 7 April 1919, 6; *West Australian*, 7 April 1919, 6; *West Australian*, 16 April 1919, 7; Telegram from Mayor of Boulder, Beston to Acting Prime Minister Watt, 17 April 1919, NAA: CP103/11 415—Influenza Epidemic. Transcontinental Railways Miscellaneous; Pneumonic Influenza leaflet, 1919, State Library of Western Australia; Letter from E. W. V. Gribble, Town Clerk of Boulder Municipality to Colebatch, 16 April 1919, SROWA, S36 Cons1496 1919/0033.

190
*Fremantle Times*, 14 March 1919, 2.

191
*West Australian*, 6 February 1919, 4.

192 Melbourne *Argus*, 12 March 1919, 9.

193
*Fremantle Times*, 14 March 1919, 2.

194
*Ibid*., 2.

195 Blackwell, *Western Isolation*, 56.

196 Spencer, *J.**H.**L. Cumpston*, 168.

197
*Ibid*.

198
*Ibid*.

199 Quoted, unsourced, in Spencer, *J.H.L. Cumpston*, 168.

200
*Daily News*, 14 April 1919, 4.

201 Telegram from Gardiner to Robinson (Minister for Industries), 24 February 1919, SROWA: S36 Cons1496 1919/0001.

202 Atkinson, *Report for the Two Years Ending*, 10.

203 ‘Influenza Epidemic, 1918’, NAA: A6006, 1919/2/3.

204 Spencer, *J.H.L. Cumpston*, 169.

205 Spencer, *J.H.L. Cumpston*, 168.

206 Communications between Watt and Lefroy, March-April 1919, SROWA, S36 Cons1496 1919/0033.

207 Telegram from H. P. Colebatch to Henry Lefroy, 23 February 1919, 21 February 1919, SROWA: S36 Cons1496 1919/0001 Premiers & Ministers Conference Melbourne Jan 1919; Atkinson, *Report for the Two Years Ending*, 10.

208
*Western Mail*, 18 April 1919, 13.

209 Hobart *Mercury*, 19 April 1919, 7.

210 Hyslop, ‘Insidious Immigrant’, 207.

211 See B. K. de Garis, ‘An Incident at Fremantle’, *Labour History*, 1966, 10, 33.

212 Letter from Robert Robinson, Minister for Industries to Colebatch, 24 February 1919, SROWA, S36 Cons1496 1919/0033.

213 Telegram from Robinson to Gardiner, 24 February 1919, Telegram from Robinson to Gardiner, 24 February 1919, SROWA: S36 Cons 1496 1919/0033 Part 1 Influenza in Eastern States.

214
*West Australian*, 21 April 1919, 6; Hon J. Mackinnon Fowler to Watt, 7 February 1919, NAA: A2, item 19/1131, quoted in Hyslop, ‘Insidious Immigrant’, 208.

215
*West Australian*, 21 April 1919, 6.

216 B. K. de Garis, ‘A Political Biography of Sir Hal Colebatch’, (unpublished Master’s thesis, University of Western Australia, 1962), 99–100.

217 Hyslop, ‘Insidious Immigrant’, 211.

218
*Official Year Book of the Commonwealth of Australia*, 1901–1913, 13 (Melbourne: Government Printer, 1929), 1129, cited in Hyslop, ‘Insidious Immigrant’, 213.

219 W. A. Watt, *Commonwealth Parliamentary Debates*, 25 June 1919.

220 Stephen Bartos, ‘Federation Balance of Power: Things Have Changed’, *Mandarin*, 28 April 2021, https://www.themandarin.com.au/154979-federation-balance-of-power-things-have-changed-part-1/; accessed 18 May 2021.

221 ‘Daniel Andrews Is Finally Taking a Day Off. Is This a Sign Things Are Looking Up for Victoria?’, *ABC News*, 30 October 2020, https://www.abc.net.au/news/2020-10-30/daniel-andrews-takes-day-off-after-120-covid-media-briefings/12831460, accessed 19 May 2021.

222 Stephen Duckett, ‘Four Ways Australia’s Vaccine Rollout Has Been Bungled’, *Conversation*, 1 April 2021, https://theconversation.com/4-ways-australias-covid-vaccine-rollout-has-been-bungled-158225; accessed 19 May 2021; David Crowe, ‘Almost One Third of Australians Say They’re Unlikely to Get COVID Vaccination: Survey’, *Sydney Morning Herald*, 19 May 2021, https://www.smh.com.au/politics/federal/almost-one-third-of-adult-australians-say-they-re-unlikely-to-get-covid-vaccination-survey-20210518-p57szo.html; accessed 19 May 2021.

## Notes

Dr Carolyn Holbrook is an Australian Research Council DECRA Fellow at Deakin University in Melbourne, Australia. She is writing a cultural history of the Australian federation and leading an international study of public health systems. She is working with Professor David Lowe on an Australian Research Council project about the conceptual history of national security in Australia since 1901. Carolyn is the Director of Australian Policy and History.

